# Targeting the integrated networks of aggresome formation, proteasome, and autophagy potentiates ER stress-mediated cell death in multiple myeloma cells

**DOI:** 10.3892/ijo.2014.2773

**Published:** 2014-11-24

**Authors:** SHOTA MORIYA, SEIICHIRO KOMATSU, KAHO YAMASAKI, YUSUKE KAWAI, HIROKO KOKUBA, AYAKO HIROTA, XIAO-FANG CHE, MASATO INAZU, AKIHIKO GOTOH, MASAKI HIRAMOTO, KEISUKE MIYAZAWA

**Affiliations:** 1 Department of Biochemistry, Tokyo Medical University, Tokyo, Japan; 2 Department of Breast Oncology, Tokyo Medical University, Tokyo, Japan; 3 Laboratory of Electron Microscopy, Tokyo Medical University, Tokyo, Japan; 4 Institute of Medical Science, Tokyo Medical University, Tokyo, Japan; 5 Department of Hematology, Juntendo University, Tokyo, Japan

**Keywords:** multiple myeloma, aggresome, proteasome, autophagy, ER stress

## Abstract

The inhibitory effects of macrolide antibiotics including clarithromycin (CAM) on autophagy flux have been reported. Although a macrolide antibiotic exhibits no cytotoxicity, its combination with bortezomib (BZ), a proteasome inhibitor, for the simultaneous blocking of the ubiquitin (Ub)-proteasome and autophagy-lysosome pathways leads to enhanced multiple myeloma (MM) cell apoptosis induction via stress overloading of the endoplasmic reticulum (ER). As misfolded protein cargo is recruited by histone deacetylase 6 (HDAC6) to dynein motors for aggresome transport, serving to sequester misfolded proteins, we further investigated the cellular effects of targeting proteolytic pathways and aggresome formation concomitantly in MM cells. Pronounced apoptosis was induced by the combination of vorinostat [suberoylanilide hydroxamic acid (SAHA); potently inhibits HDAC6] with CAM and BZ compared with each reagent or a 2-reagent combination. CAM/BZ treatment induced vimentin positive-aggresome formation along with the accumulation of autolysosomes in the perinuclear region, whereas they were inhibited in the presence of SAHA. The SAHA/CAM/BZ combination treatment maximally upregulated genes related to ER stress including C/EBP homologous protein (CHOP). Similarly to MM cell lines, enhanced cytotoxicity with CHOP upregulation following SAHA/CAM/BZ treatment was shown by a wild-type murine embryonic fibroblast (MEF) cell line; however, a CHOP-deficient MEF cell line almost completely canceled this pronounced cytotoxicity. Knockdown of HDAC6 with siRNA exhibited further enhanced CAM/BZ-induced cytotoxicity and CHOP induction along with the cancellation of aggresome formation. Targeting the integrated networks of aggresome, proteasome, and autophagy is suggested to induce efficient ER stress-mediated apoptosis in MM cells.

## Introduction

Multiple myeloma (MM) is a refractory hematopoietic malignancy showing clonal plasma cell accumulation. A major breakthrough in MM treatment has been the introduction of the first-in-class proteasome inhibitor bortezomib (BZ) (1,2). Moreover, the treatment of relapsed and refractory MM is now possible with carfilzomib, a second-generation proteasome inhibitor, and immunomodulatory agents. This has offered new alternatives for vulnerable patients (3,4). However, patients with relapsed and refractory MM are still urgent issues and require treatment combinations (5,6). Increasing lines of evidence indicate that proteasome inhibition induces misfolded protein accumulation in the endoplasmic reticulum (ER) (7,8). This evokes ER stress followed by the unfolded protein response (UPR) (9,10). UPR mainly functions: i) to decrease protein entry into the ER by suppressing translational rate; and ii) to increase the folding capacity of the ER via chaperon protein translational activations. Following incorrect folding in the ER, proteins are retro-translocated for degradation in the cytoplasm via the ubiquitin (Ub)-proteasome pathway [i.e., ER-associated degradation (ERAD)]. A failure in all the adaptation strategies triggers apoptosis and induces C/EBP homologous protein (CHOP) (GADD153), a pro-apoptotic transcription factor and other pathways (7–10). Since MM is characterized by the uncontrolled cell growth of monoclonal antibody (mAb)-producing plasma cells, production of large quantities of unfolded or misfolded immunoglobulin triggers ER stress. Thus, therapeutic manipulation of the UPR pathway appears to disrupt cellular mechanisms for processing high protein loads and cellular stress, and further leads to death of MM cells.

Macroautophagy (hereafter, ‘autophagy’) occurs when cellular proteins and organelles (e.g., ER) are enveloped in an autophagosome and degraded in lysosomes by lysosomal hydrolases (11,12). Although autophagy is considered a bulk non-selective degradation of long-lived proteins and organelles, recent reports revealed the selective degradation pathway of ubiquitinated protein via autophagy using docking proteins (e.g., p62) and related proteins (e.g., NBR1), having both a microtubule-associated protein 1 light chain 3 (LC3)-interacting region and a Ub-associated domain (13,14). Thus, overflowed ubiquitinated proteins are bound to p62 and subsequently engulfed into an autophagosome via the LC3-interacting region in p62. This indicates that autophagy also acts as a compensatory degradation system when the proteasome system is impaired (14). We previously reported that the inhibition of autophagy using the autophagy inhibitor bafilomycin A_1_ (BAF) enhanced BZ-induced apoptosis by burdening ER stress in MM cell lines (15). We also reported that macrolide antibiotics [e.g., clarithromycin (CAM) and azithromycin (AZM)] attenuated or blocked autophagy flux, possibly mediated through the inhibition of the lysosomal function, and that ER stress loading was enhanced by both the BZ-induced inhibition of the Ub-proteasome system and the CAM- or AZM-induced inhibition of the autophagy-lysosome system. This is followed by CHOP transcriptional activation and the induction of apoptosis in MM and breast cancer cells (16,17). Therefore, concomitant blocking of proteasome and autophagy appears to be a promising combination therapy.

Moreover, misfolded/unfolded proteins are sequestrated into aggregates and transported. They are then discarded from the cytoplasm by dynein motors through the microtubule network to the aggresome (18). The class II histone deacetylase 6 (HDAC6), which is a microtubule-associated deacetylase and an aggresome component, has the capacity to bind both polyubiquitinated misfolded proteins and dynein motors (19). By acting as an adaptor between ubiquitinated protein aggregates and dynein, HDAC6 enables aggregated protein loading onto the dynein motor protein complex (20). Thus, in the formation of aggresome at the microtubule-organizing center (MTOC), HDAC6, dynein, and polyubiquitinated proteins functionally interact with each other (19,20). If HDAC6 is lacking, cells will not be able to clear cytoplasmic unfolded protein aggregates; cells cannot appropriately form aggresomes; and cells become hypersensitive to unfolded protein accumulation (19). Therefore, HDAC6 appears to be another critical factor in the management of unfolded protein-induced stress at the cellular level. Moreover, some parts of the aggresome are reported to be degraded via the autophagy-lysosome system (21,22). A recent study also revealed that p62 regulates the accumulation and autophagic clearance of protein aggregates by directly binding to HDAC6, and this interaction appears to regulate HDAC6 activity (23).

All these lines of evidence suggest the integrated intracellular networks of proteasome, autophagy, and aggresome for unfolded protein processing. ER stress loading may be further enhanced by targeting both intracellular proteolytic pathways and aggresome formation (24). To prove this hypothesis aimed for clinical application, we intentionally attempted to use well-approved drugs such as CAM, BZ, and vorinostat [suberoylanilide hydroxamic acid (SAHA)], an orally bioavailable inhibitor of HDAC with a half maximal inhibitory concentration (IC_50_) of 37 nM and which has passed the assessment of the Food and Drug Administration for cutaneous T-cell lymphoma treatment (25,26). In the present investigation, we clearly demonstrated that the SAHA/CAM/BZ combination treatment induced marked ER stress-mediated MM cell death. This provides a promising treatment for MM patients in the form of ‘ER stress-loading therapy’.

## Materials and methods

### Reagents

BZ was purchased from Selleck Chemicals (Houston, TX, USA). CAM was purchased from Tokyo Chemical Industry Co., Ltd. (Tokyo, Japan), SAHA was from Cayman Chemical Co. (Ann Arbor, MI, USA), and tubacin was from Sigma-Aldrich (St. Louis, MO, USA). BZ, SAHA and tubacin were dissolved in dimethyl sulfoxide to make stock solutions at concentrations of 1, 10 and 1 mM, respectively. CAM was dissolved in ethanol to prepare stock solutions of 5 mg/ml.

### Cell lines and culture conditions

For this study, the MM cell lines IM-9 and RPMI-8226, and the lung carcinoma cell line H226 were obtained from the American Type Culture Collection (ATCC) (Manassas, VA, USA). The human MM cell line KMS-12-PE was obtained from the Japanese Collection of Research Bioresources (JCRB) (Osaka, Japan). A CHOP^−/−^ murine embryonic fibroblast (MEF) cell line (CHOP-KO-DR) established from a 13.5-day-old CHOP^−/−^ mouse embryo by SV-40 immortalization and a CHOP^+/+^ MEF cell line (DR-wild-type) established by SV-40 immortalization as a control cell line for CHOP-KO-DR were also obtained from the ATCC. These authorized cell lines were expanded and frozen in aliquots within 1 month after obtaining from the cell banks. Each aliquot was thawed and the cells were used for the experiments within 2 months after thawing. IM-9, H226, RPMI-8226, and KMS-12-PE cells were cultured in RPMI-1640 medium (Sigma-Aldrich) supplemented with 10% fetal bovine serum (FBS) (Biowest SAS, Nuaillé, France), 2 mM L-glutamine, penicillin (100 U/ml), and streptomycin (100 μg/ml) (Wako Pure Chemicals Industries, Tokyo, Japan). CHOP-KO-DR and DR-wild-type cells were maintained in Dulbecco’s modified Eagle’s medium (Sigma-Aldrich) supplemented with 10% FBS, 2 mM L-glutamine, penicillin (100 U/ml), and streptomycin (100 μg/ml). All cell lines were cultured in a humidified incubator containing 5% CO_2_ and 95% air at 37°C.

### Assessment of viable number of cells

The number of viable cells was assessed using CellTiter-Blue Cell Viability Assay kit (Promega Corp., Madison, WI, USA) according to the manufacturer’s instructions as previously described in detail (16).

### Immunoblotting

Immunoblotting was performed as previously described (15,16). In brief, cells were lysed with RIPA lysis buffer supplemented with a protease and phosphatase inhibitor cocktail (both from Nacalai Tesque, Kyoto, Japan). Cellular proteins were quantified using a DC Protein Assay kit (Bio-Rad, Hercules, CA, USA). Equal amounts of proteins were loaded onto the gels, separated by sodium dodecyl sulfate polyacrylamide gel electrophoresis (SDS-PAGE), and transferred onto Immobilon-P membrane (Millipore, Billerica, MA, USA). The membranes were probed with primary antibodies (Abs) such as anti-acetylated-α-tubulin (6–11B-1) mAb, anti-α-tubulin (B-7) mAb, anti-GAPDH (6C5) mAb, anti-HDAC6 (H-300) Ab, and anti-Ub (P4D1) mAb, which were all purchased from Santa Cruz Biotechnology, Inc. (Santa Cruz, CA, USA) and anti-PARP Ab (Cell Signaling Technology, Inc., Danvers, MA, USA). Immunoreactive proteins were detected with horseradish peroxidase-conjugated secondary Abs (Cell Signaling Technology, Inc.) and an enhanced chemiluminescence reagent (Millipore). Densitometry was performed using a Molecular Imager ChemiDoc XRS System (Bio-Rad).

### RNA interference

For the gene silencing of HDAC6 in RPMI-8226 and H226 cells, HDAC6 siRNA and control siRNA were purchased from Life Technologies (Grand Island, NY, USA) and whose sequences are described as follows: HDAC6 sense, CCAGCACAGUCUUAUGGAUGCUAU and antisense, AUAGCCAUCCAUAAGACUGUGCUGG. siRNAs were diluted to a final concentration of 33 nM in Opti-MEM I (Life Technologies). Transfection was performed with the cells at 40% confluency using Lipofectamine RNAiMAX transfection reagent (Life Technologies) according to the manufacturer’s instructions. Knockdown efficiency was assessed by immunoblotting.

### Gene expression analysis

Total RNA was isolated from cell pellets using Isogen (Wako Pure Chemicals Industries) and genomic DNA was removed using RQ1 RNase-Free DNase (Promega Corp.) at 37°C for 30 min, followed by extraction with phenol chloroform and ethanol precipitation. Reverse-transcription using a PrimeScript RT Master Mix (Takara Bio, Inc., Shiga, Japan) was performed according to the manufacturer’s instructions. Real-time polymerase chain reaction (PCR) was performed on 3 ng of cDNA using validated SYBR-Green gene expression assays for human ER stress-related genes (*CHOP*, *BAX*, *BIM*, *DR5*, *GADD34* and *GRP78*) in combination with SYBR Premix Ex Taq II (Takara Bio, Inc.). The sequences of primers and reaction conditions were previously described (16). Quantitative real-time PCR was performed in duplicates in a Thermal Cycler Dice Real-Time System TP800 (Takara Bio, Inc.). The data were analyzed using Thermal Cycler Dice Real-Time System Software version 5.00 (Takara Bio, Inc.), and the comparative Ct method (2^−ΔΔCt^) was used for the relative quantification of gene expression. The data of real-time PCR products were standardized to *GAPDH* as an internal control. To confirm the specific amplification of target genes, each gene product was further separated by using 1.5% agarose gel after real-time PCR to detect a single band at the theoretical product size, as well as by analysis of the dissociation curve for detecting a single peak.

### Immunocytochemistry and confocal microscopy

Cells were spread on slide glasses using Cytospin 4 Centrifuge (Thermo Fisher Scientific, Inc., Rockford, IL, USA) to make slide glass preparations. Cells were fixed for 20 min in ice-cold methanol and permeabilized with 0.1% Triton X-100 for 20 min, followed by blocking with 2% bovine serum albumin in TBST (25 mM Tris, 137 mM NaCl, 2.7 mM KCl, 0.05% Tween-20, pH 7.4) for 1 h. Cells were immunostained with primary Abs such as mouse anti-vimentin (V9) mAb, mouse anti-Ub mAb, and lysosomal-associated membrane protein-1 (LAMP-1) (H4A3) mAbs (all from Santa Cruz Biotechnology, Inc.). The secondary Abs used for fluorescence detection were Alexa Fluor^®^ 488 F(ab′)2 fragment of goat anti-mouse IgG (H+L) Ab (Life Technologies). Nuclei were stained with 4′,6-diamidino-2-phenylindole (DAPI) (Wako Pure Chemicals Industries). Slides were mounted with SlowFade Gold antifade reagent (Life Technologies). Analysis by confocal microscopy was performed using the confocal laser scanning fluorescence microscope FV10i-DOC.(Olympus Corp., Tokyo, Japan).

### Assessment of aggresome by fractionation of detergent-soluble and -insoluble proteins

Cells were lysed with Triton X-100 lysis buffer (10 mM Tris-HCl, 150 mM NaCl, 2% Triton X-100, pH 7.8) supplemented with a protease inhibitor cocktail (Nacalai Tesque). The lysates were centrifuged at 12,000 g for 30 min at 4°C. The supernatant was then collected as the soluble fraction. The pellets (which contain the insoluble protein) were then resuspended in sodium dodecyl sulfate (SDS) lysis buffer (10 mM Tris-HCl, 150 mM NaCl, 2% SDS, pH 7.8) and sonicated for 30 sec with a tip sonicator VP-5S (Taitec, Saitama, Japan) to prepare the insoluble fraction. Equal volumes of each pellet and supernatants were boiled for 5 min in SDS-PAGE sample buffer (125 mM Tris-HCl, 4% SDS, 20% glycerol, 0.002% BPB, pH 6.8) and analyzed by SDS-PAGE.

### Electron microscopy

Cells were fixed with 2.5% glutaraldehyde in 0.1 M phosphate buffer (pH 7.4) for 1 h. The samples were further fixed in 1% osmium tetroxide for 1 h, dehydrated in graded ethanol (30–100%), and embedded in Quetol 812 epoxy resin (Nisshin EM Co., Ltd., Tokyo, Japan). Ultrathin sections were cut with an Ultracut J microtome (Reichert-Jung, Vienna, Austria). The sections were stained with lead nitrate and uranium acetate, and subjected to electron microscopic analysis using the scanning electron microscope JEM-1200 EXII (JEOL, Tokyo, Japan).

### Statistical analysis

All data are expressed as mean ± SD. Statistical analysis was performed using Mann-Whitney U test (two-tailed).

## Results

### SAHA, BZ, and CAM combination treatment potently enhanced MM cell apoptosis

**Figure 1:**
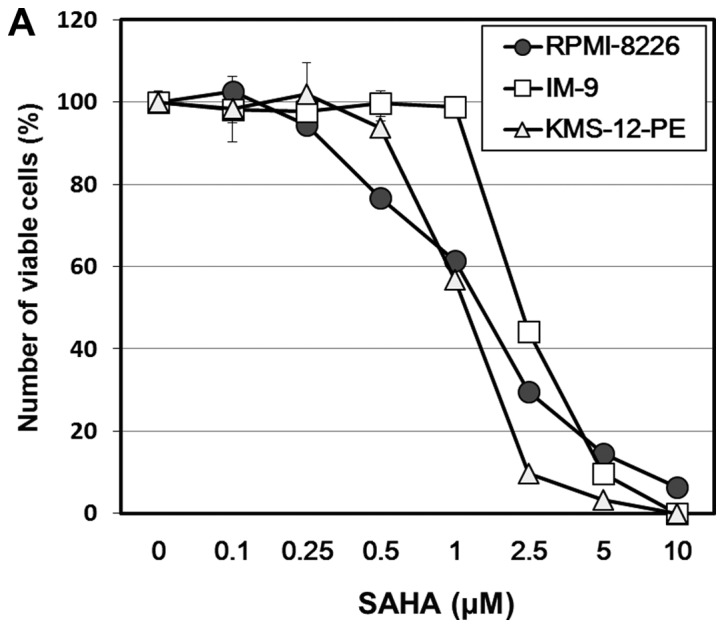

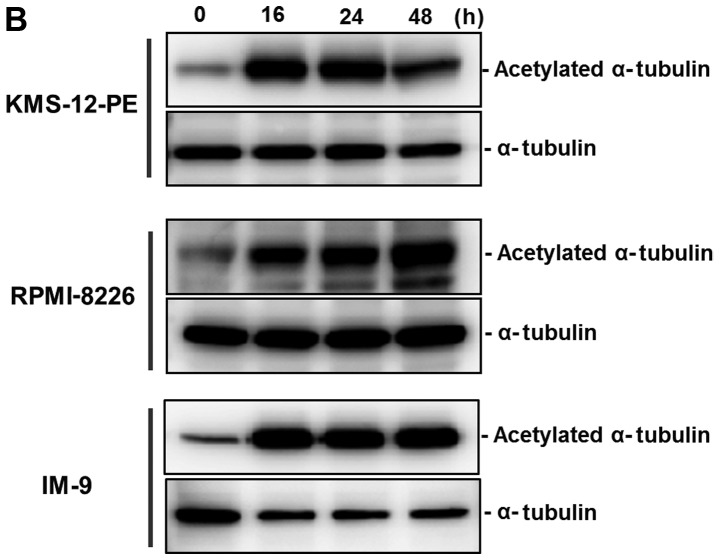
Cell growth inhibition and α-tubulin acetylation after treatment with suberoylanilide hydroxamic acid (SAHA) in myeloma cell lines. (A) The myeloma cells KMS-12-PE, RPMI-8226, and IM-9 were cultured in the presence of SAHA at various concentrations for 48 h. Cell growth inhibition was assessed as described in Materials and methods. (B) After treatment with SAHA (at 0.5 μM in KMS-12-PE, RPMI-8226, and at 1.0 μM in IM-9) for 16–48 h, cellular proteins were lysed, separated by 11.25% sodium dodecyl sulfate polyacrylamide gel electrophoresis (SDS-PAGE), and immunoblotted using a specific monoclonal antibody (mAb) against the acetylated α-tubulin. Immunoblotting with anti-α-tubulin mAb was performed as an internal control.

**Figure 2:**
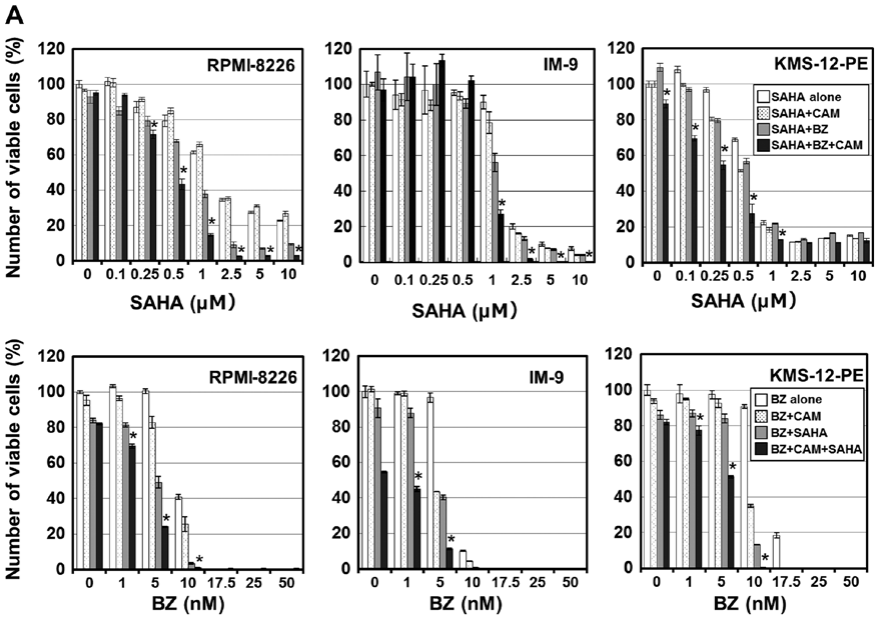

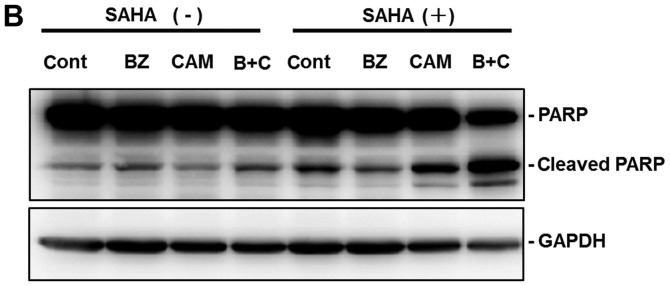
Cell growth inhibition after treatment with suberoylanilide hydroxamic acid (SAHA), bortezomib (BZ), and clarithromycin (CAM) in myeloma cell lines. (A) Upper panels: myeloma cell lines RPMI-8226, IM-9, and KMS-12-PE were cultured in the presence of various concentrations of SAHA with/without either BZ (5 nM) or CAM (50 μg/ml) for 48 h. Lower panels: myeloma cell lines were cultured in the presence of various concentrations of BZ with/without either SAHA (at 0.5 μM in KMS-12-PE, RPMI-8226, and at 1.0 μM in IM-9) or CAM (50 μg/ml) for 48 h. Viable cell numbers were assessed as described in Materials and methods. ^*^P<0.05 SAHA/BZ/CAM vs. SAHA/BZ and SAHA/CAM and BZ/CAM. (B) IM-9 cells were treated with either BZ (5 nM) or CAM (50 μg/ml) in the presence or absence or SAHA (1 μM) for 48 h. Cellular proteins were separated by 11.25% sodium dodecyl sulfate polyacrylamide gel electrophoresis (SDS-PAGE), and immunoblotted with anti-PARP monoclonal antibody (mAb). Immunoblotting with GAPDH mAb was performed as an internal control for protein loading.

Treatment with SAHA for 48 h resulted in a dose-dependent inhibition of cellular growth in all MM cell lines (Fig. 1A). The IC_50_ was 1.2 μM in KMS-12-PE, 1.5 μM in RPMI-8226, and 2.1 μM in IM-9 cells. SAHA-treated MM cells exhibited apoptotic morphologic features such as fragmentation of the nucleus and formation of an apoptotic body along with cleavage of PARP and caspase-3 (data not shown). The regulation of the stability and function of a microtubule has been associated with α-tubulin reversible acetylation, and HDAC6 functions as α-tubulin deacetylase (27). Immunoblotting using a specific Ab for the acetylated α-tubulin revealed that, in response to SAHA (at 0.5 μM for KMS-12-PE and RPMI-8226, at 1 μM for IM-9), the acetylation of α-tubulin was detectable within 16 h and persisted for at least 48 h in all three cell lines tested (Fig. 1B). In our previous studies, autophagy flux was shown to be blocked by CAM, and that the BZ and CAM combination treatment resulted in ER-stress overloading followed by enhanced induction of apoptosis in MM and breast cancer cells (16,17). Since HDAC6 has been implicated in aggresome formation that results in sequestering overabundant intracellular unfolded proteins (18), we examined whether the SAHA plus BZ and/or CAM combination treatment further increased cytotoxicity via ER stress loading. As shown in Fig. 2A, the combined treatment with SAHA/BZ, but not with SAHA/CAM, potentiated cell growth inhibition. Notably, although CAM treatment alone at 50 μg/ml did not inhibit the growth of cells, the combined SAHA/BZ/CAM treatment showed a clearly pronounced cytotoxicity compared with the SAHA/BZ or BZ/CAM treatment in all MM cell lines. This enhanced cytotoxicity was mediated through apoptosis induction since the pronounced expression of the cleaved PARP was observed in response to the suboptimal concentrations of the SAHA/BZ/CAM combination in IM-9 cells (Fig. 2B).

**Figure 3:**
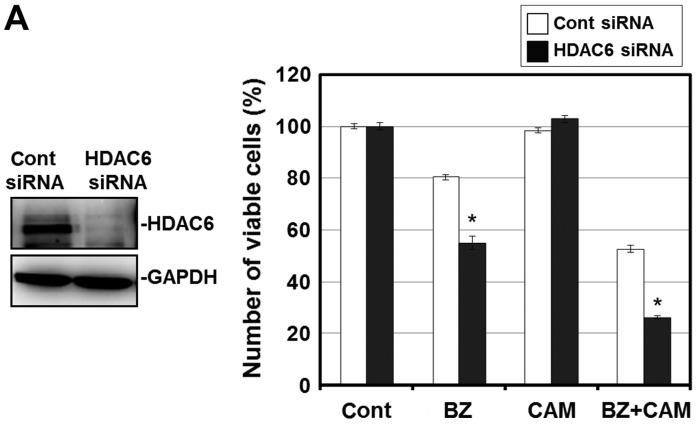

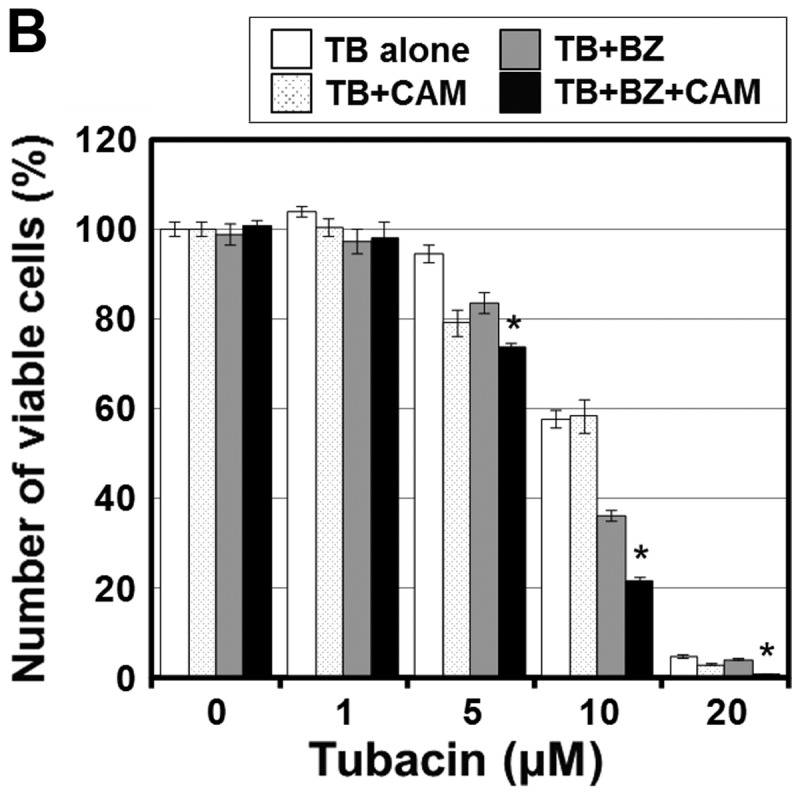
Effects of histone deacetylase 6 (HDAC6) inhibition on myeloma cell growth. (A) Suppression of HDAC6 expression using HDAC6 siRNA : RPMI-8226 cells were pre-treated with either HDAC6 siRNA or control siRNA for 48 h. Subsequently, the cells were cultured in the presence or absence of bortezomib (BZ) (10 nM) with/without clarithromycin (CAM) (50 μg/ml) for 48 h. ^*^P<0.05 cont siRNA vs. HDAC6 siRNA. (B) Enzymatic inhibition of HDAC6 with a specific HDAC6 inhibitor, tubacin: RPMI-8226 cells were treated with tubacin at indicated concentrations in the presence or absence of either BZ (5 nM) or CAM (50 μg/ml) for 48 h. Viable cell numbers were assessed as described in Materials and methods. ^*^P<0.05 TB/BZ/CAM vs. TB/BZ and TB/CAM.

To confirm that this enhanced cytotoxicity is mediated through HDAC6 inhibition, we next attempted to knock down HDAC6 with siRNA. Pre-treatment with HDAC6 siRNA effectively suppressed the expression level of HDAC6 in RPMI-8226 cells. Under this condition, the cell growth inhibition by the BZ/CAM treatment was pronounced compared with the cells pre-treated with control siRNA. In addition, tubacin, a specific HDAC6 inhibitor, reproduced the pronounced cytotoxicity when combined with BZ and BZ/CAM (Fig. 3A and B) (28). These results suggest that HDAC6 inhibition appears to be involved in the enhancement of cytotoxicity induced by the addition of SAHA to BZ/CAM-containing cell culture medium.

### SAHA suppressed the aggresome formation induced by simultaneous inhibition of proteasome and autophagy in MM cells

**Figure 4:**
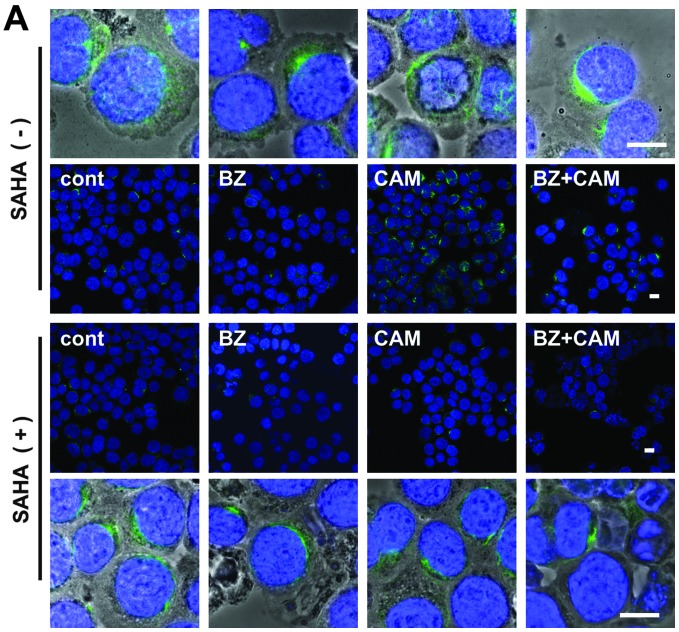

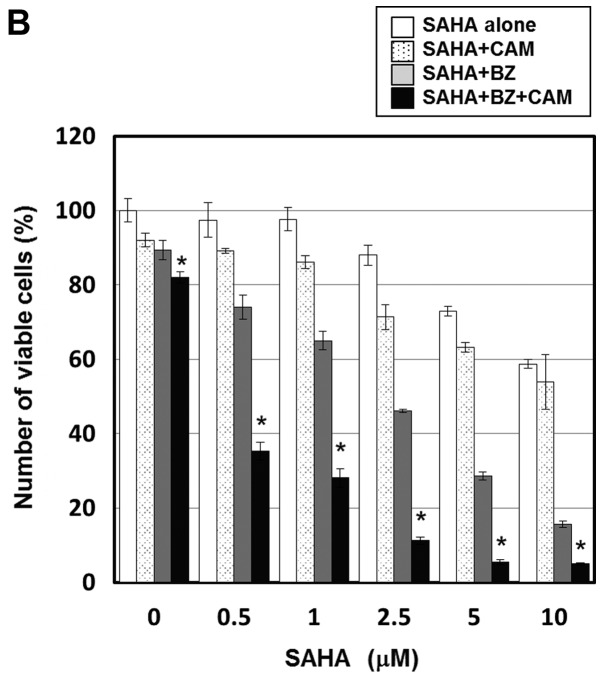

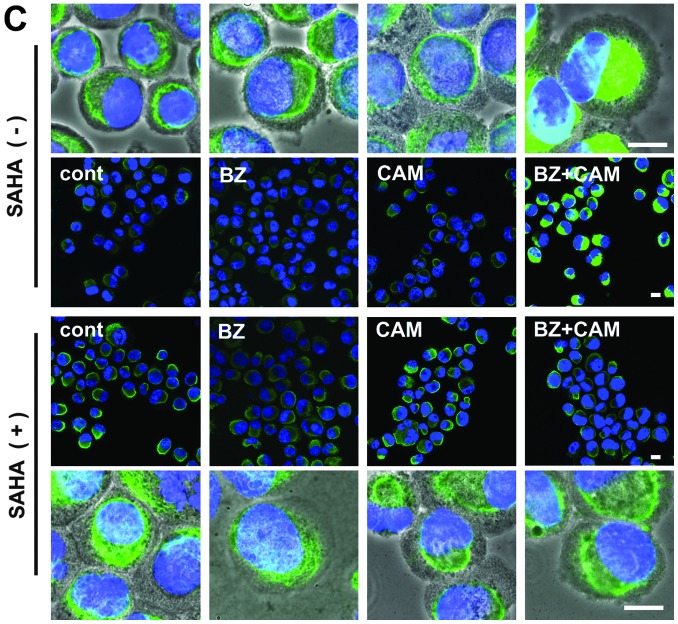

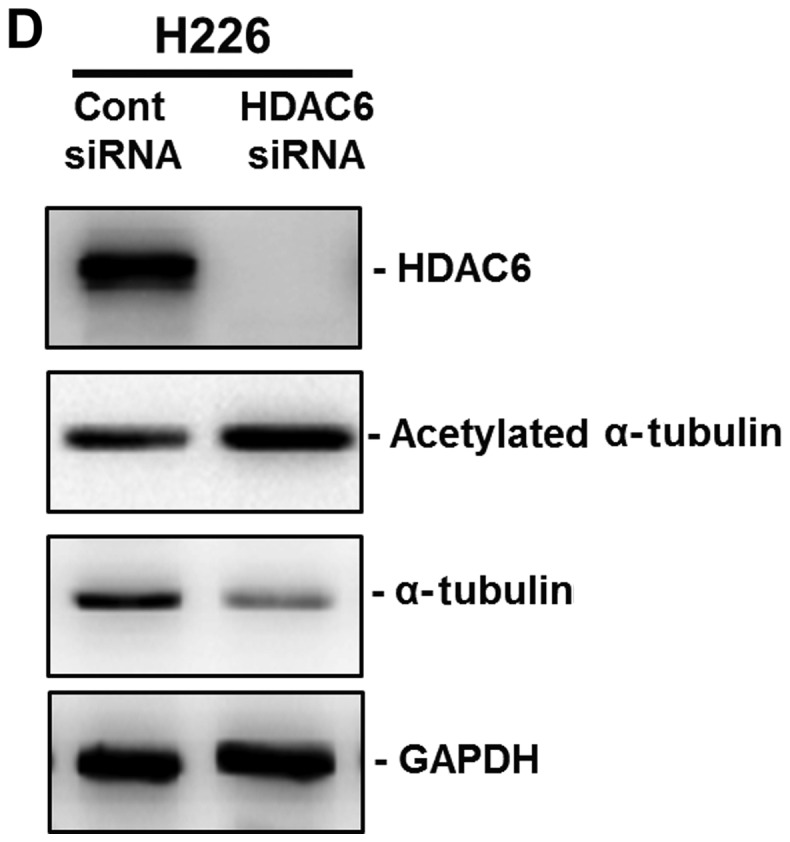

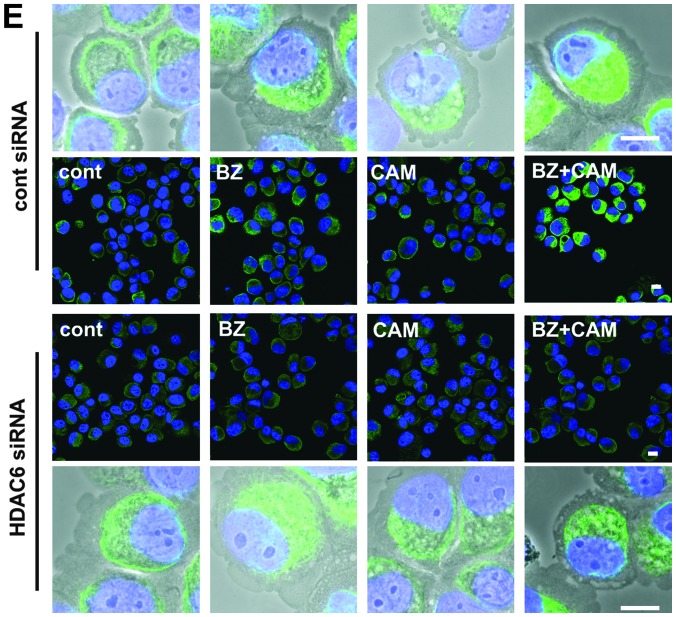

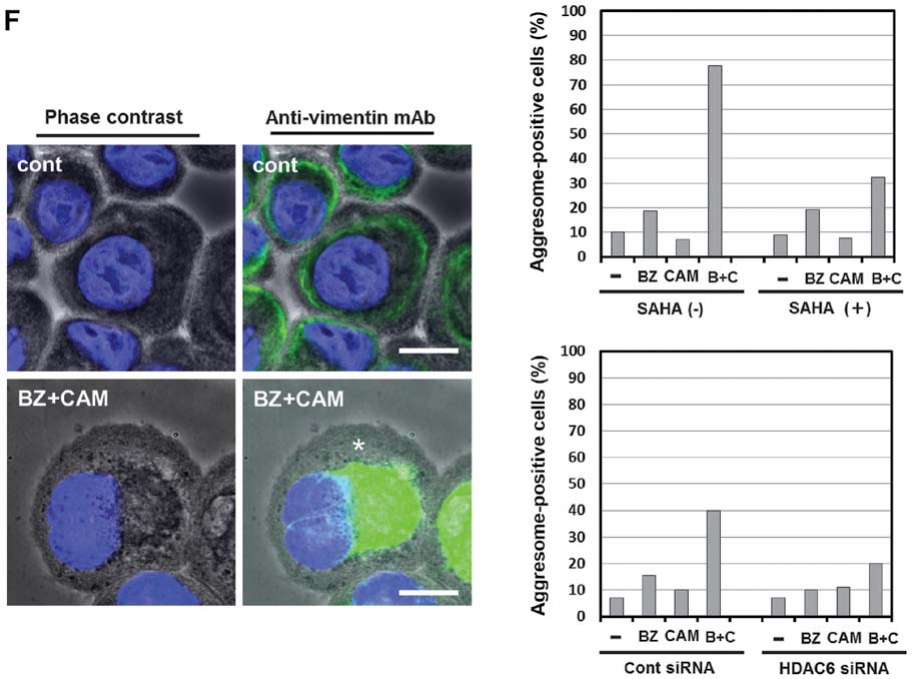
Assessment of aggresome formation by immunocytochemistry using anti-vimentin monoclonal antibody (mAb). (A) RPMI-8226 cells were treated with either bortezomib (BZ) (5 nM) or clarithromycin (CAM) (50 μg/ml) in the presence/absence of suberoylanilide hydroxamic acid (SAHA) (0.5 μM) for 16 h. Immunocytochemistry using anti-vimentin mAb was performed. 4′,6-diamidino-2-phenylindole (DAPI) staining shows the position of nucleus (blue). (B) H226 cells were cultured in the presence of various concentrations of SAHA with/without either BZ (5 nM) or CAM (50 μg/ml) for 48 h. Viable cell numbers were assessed as described in Materials and methods. ^*^P<0.05 SAHA/BZ/CAM vs. SAHA/BZ and SAHA/CAM. (C) H226 cells were treated with either BZ (5 nM) or CAM (50 μg/ml) in the presence/absence of SAHA (0.5 μM) for 24 h. Immunocytochemistry using anti-vimentin mAb was performed. (D) H226 cells were pre-treated with histone deacetylase 6 (HDAC6) siRNA or control siRNA for 48 h. Then, the expression levels of HDAC6 and acetylated α-tubulin were assessed by immunoblotting. (E) After pre-treatment with siRNA, H226 cells were further exposed to BZ (5 nM) and/or CAM (50 μg/ml) for 24 h. Immunocytochemistry was performed using anti-vimentin mAb. Assessment of aggresome formation by immunocytochemistry using anti-vimentin monoclonal antibody (mAb). (F) Percentage of ‘aggresome-positive’ cells in H226 cells after treatment with either BZ±CAM±SAHA or BZ±CAM pre-treated with HDAC6 siRNA or control siRNA. One hundred cells stained with anti-vimentin mAb were assessed for their staining pattern. The cells with high dense staining in the perinuclear region (indicated by ^*^ in the left lower panel) were defined and plotted as ‘aggresome-positive’ cells. Each scale bar indicates 10 μm.

To further confirm that the pronounced cytotoxicity by SAHA shown in Fig. 2A was due to the inhibition of HDAC6 activity involved in aggresome formation, we assessed aggresome formation in the presence or absence of SAHA. In RPMI-8226 cells, immunocytochemistry using anti-vimentin mAb showed that the combined treatment with BZ/CAM induced a dense deposit of vimentin in the perinuclear region, which is a well-known marker for aggresome (29). This vimentin deposit was clearly suppressed in the presence of SAHA (Fig. 4A). However, since the MM cell lines including RPMI-8226 were very sensitive to the SAHA/BZ/CAM treatment, in which apoptosis was induced within 18 h of exposure to these reagents (data not shown), precise analysis of aggresome formation was practically difficult using MM cells. We therefore used the lung squamous carcinoma cell line H226 which is less sensitive, but shows pronounced cytotoxicity in response to the SAHA/BZ/CAM combination similarly to MM cell lines (Fig. 4B). As shown in Fig. 4C, the prominent vimentin deposit in the perinuclear region was observed after combined treatment with BZ/CAM for 24 h. The vimentin deposit with high fluorescent intensity was evidently suppressed in the presence of SAHA, indicating that SAHA inhibits BZ/CAM-induced aggresome formation (Fig. 4C). Similarly to SAHA, treatment with HDAC6 siRNA suppressed the vimentin condensation in the perinuclear region in H226 cells (Fig. 4D–F).

**Figure 5:**
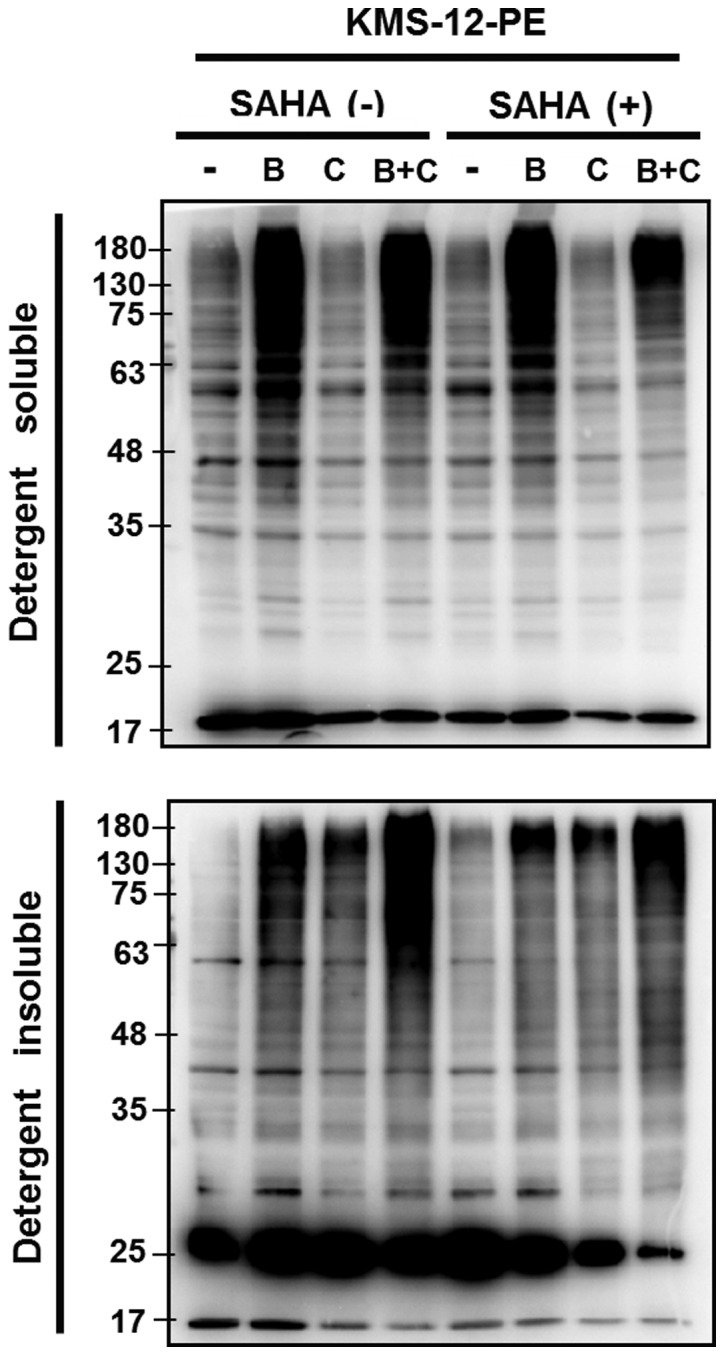
Assessment of aggresome formation by immunoblotting using anti-ubiquitin (Ub) monoclonal antibody (mAb). KMS-12-PE cells were treated with either bortezomib (B) (5 nM) or clarithromycin (C) (50 μg/ml) in the presence/absence of suberoylanilide hydroxamic acid (SAHA) (0.5 μM) for 16 h. Then cellular proteins were lysed with lysis buffer containing 2% Triton X-100 for 30 min at 0°C and followed by centrifugation at 14,000 × g for 30 min. The supernatants were used for ‘soluble fractions’. Cell pellets were resuspended in buffer containing 2% sodium dodecyl sulfate (SDS) and sonicated for 30 sec and further used as ‘insoluble fraction’. Each fractionated protein was loaded and separated by 11.25% sodium dodecyl sulfate polyacrylamide gel electrophoresis (SDS-PAGE), and immunoblotted with anti-Ub mAb.

In addition, immunocytochemistry using anti-Ub Ab exhibited the clustering of Ub-positive dots in the perinuclear region by BZ/CAM, whereas they were dispersed in the presence of SAHA in H226 cells (data not shown). As an alternative assessment of aggresome, we treated KMS-12-PE with BZ, CAM, or BZ/CAM in the presence or absence of SAHA. The cells were then treated with lysis buffer containing 2% Triton X-100, and cellular proteins were subsequently fractionated into the detergent-soluble and the -insoluble fraction. The detergent-insoluble fraction (cell pellets) was sonicated for 30 sec in the presence of 2% SDS and boiled for 5 min. Thereafter, the samples were loaded on the gels, separated by 11.25% SDS-PAGE, and immunoblotted with anti-Ub Ab. The samples derived from the detergent-soluble fractions were processed similarly. As shown in Fig. 5, the increased intracellular polyubiquitinated proteins were detectable after treatment with BZ and BZ/CAM, but not with CAM or SAHA in the detergent-soluble fraction (upper panel). In the detergent-insoluble fraction (lower panel) at higher molecular weights, prominent polyubiquitinated proteins were detected as aggresome contents in the cells treated with BZ/CAM. Treatment with BZ alone also induced accumulation of the detergent-insoluble polyubiquitinated proteins but to a lesser extent than that induced by treatment with BZ/CAM. It was noteworthy that the increased ubiquitinated proteins in the detergent-insoluble fraction were suppressed in the presence of SAHA. This also indicates the inhibition of aggresome in response to SAHA.

**Figure 6:**
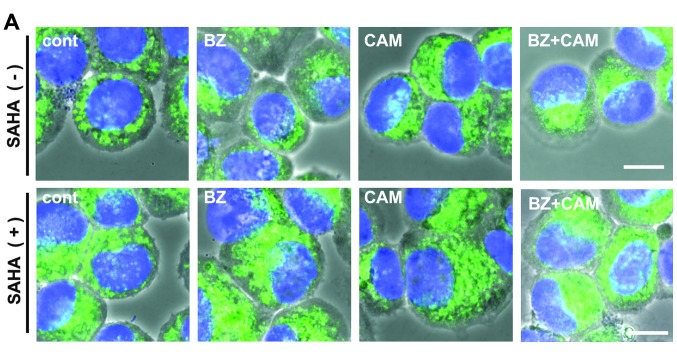

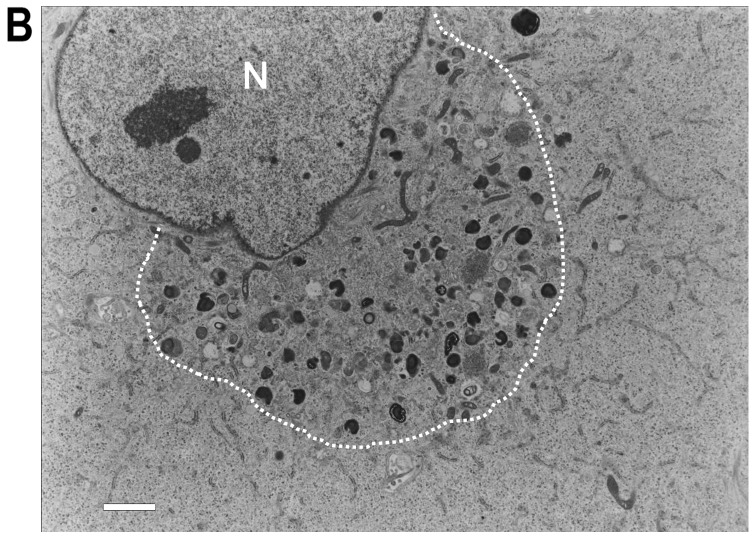

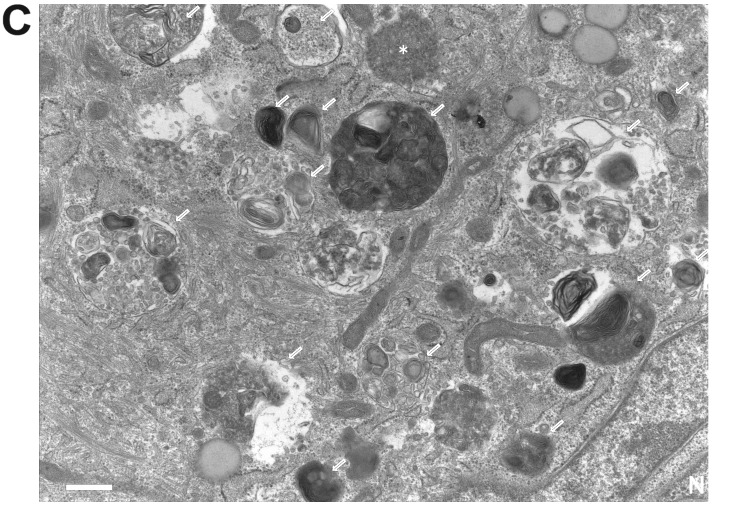

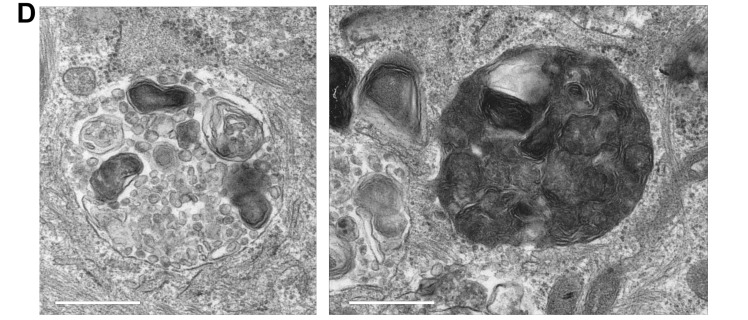
Lysosome positioning after treatment with bortezomib (BZ) and/or clarithromycin (CAM) in the presence or absence of suberoylanilide hydroxamic acid (SAHA) in H226 cells. (A) H226 cells were treated with BZ and/or CAM in the presence or absence of SAHA for 24 h. Then, immunocytochemistry was performed using anti-LAMP-1 monoclonal antibody (mAb) for detecting lysosome positioning. Merged images with phase contrast, 4′,6-diamidino-2-phenylindole (DAPI) (blue), and lysosomal-associated membrane protein-1 (LAMP-1) (green). Each scale bar indicates 10 μm. (B–D) Electron microscopy of H226 cells after treatment with BZ/CAM. (B) The region surrounded by the dashed line shows the clustering of organelles including lysosomes and mitochondria. (C) The arrows indicate autophagosomes and autolysosomes. The asterisk (^*^) appears to represent a precursor form of aggresome. (D) Clustering autolysosomes/lysosomes might represent ‘lysophagy’ (30). N, nucleus. Each scale bar indicates (B) 2 μm and (C and D) 500 nm.

Immunocytochemistry using anti-LAMP-1 mAb showed lysosome accumulation in the perinuclear region along with aggresome formation after BZ/CAM treatment. As is the case of vimentin-positive aggresome formation, the clustering of LAMP-1-positive dots was suppressed in the presence of SAHA (Fig. 6A). We therefore performed electron microscopy for precise analysis of the cytosolic area strongly stained with anti-vimentin Ab. However, contrary to what we had expected, electron microscopy exhibited no typical features of the aggresome structure such as massive accumulation of closely packed electron-dense particles surrounded by a cage of bundles of intermediated filaments in the region of the centrosome, which was first described by Johnston *et al* in 1998 (30). Instead, there was the clustering of a number of lysosomes, autophagosomes, autolysosomes, and mitochondria with increased filaments in response to the BZ/CAM treatment (Fig. 6B). A few numbers of unstructured particles with a rather high density, which might be the aggresome precursor, were detectable in this area (Fig. 6C). It was noteworthy that many lysosomal fusions or lysosomal aggregates in various sizes were observed. These might represent the phenomenon termed ‘lysophagy’ which has recently been reported by others, that is, the lysosome engulfed into an autophagosome or autolysosome (Fig. 6D) (31). In the presence of SAHA, the clustering of these organelles in the perinuclear region was suppressed to some extent along with the decreased filamentous structures. However, an increased number of lysosomes including autolysosomes was still observed compared with the cells treated with either CAM or BZ alone (Fig. 6A).

### Concomitant aggresome, proteasome, and autophagy targeting enhanced ER stress loading

**Figure 7:**
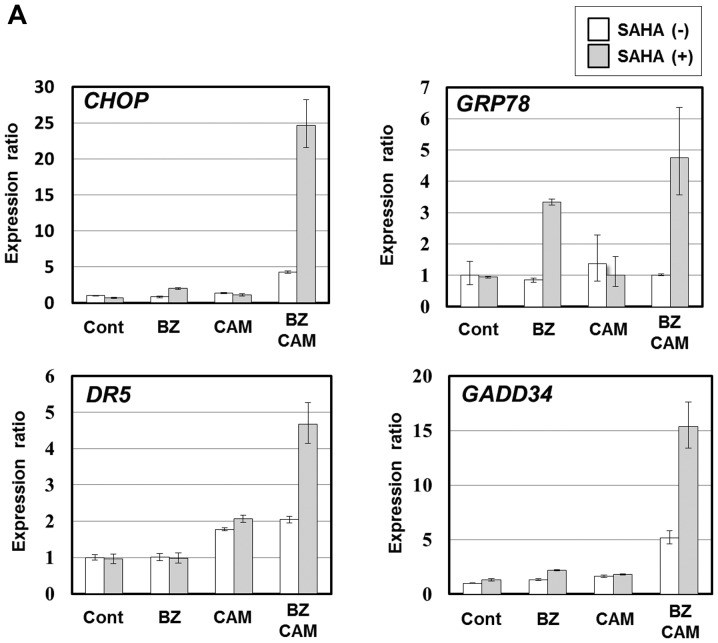

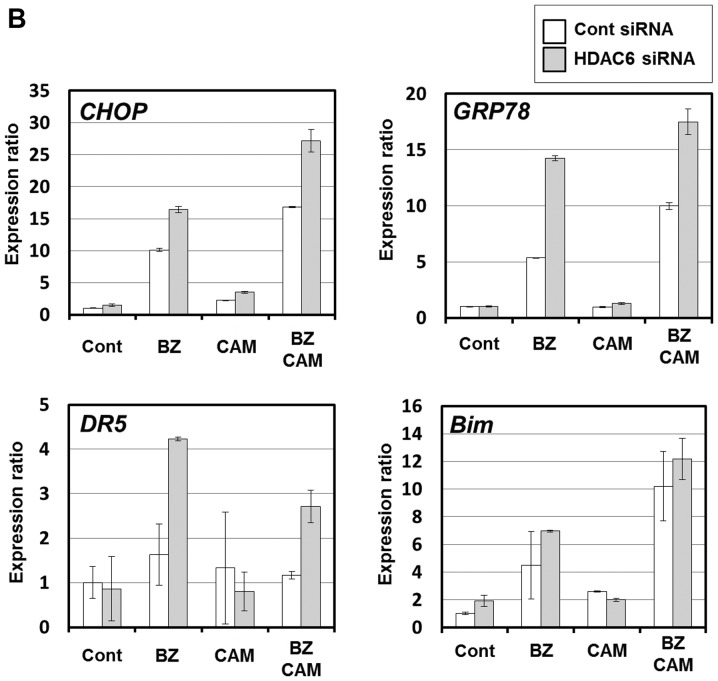

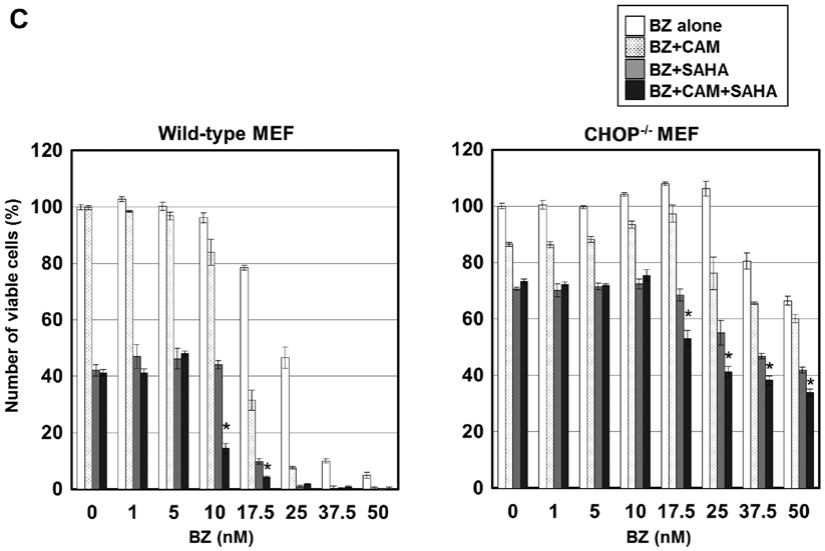
Endoplasmic reticulum (ER) stress loading after combined treatment with suberoylanilide hydroxamic acid (SAHA), bortezomib (BZ), and clarithromycin (CAM). (A) IM-9 cells were treated with BZ (5 nM) and/or CAM (50 μg/ml) in the presence or absence of SAHA (1 μM) for 16 h. Gene expressions related to ER stress were assessed by real-time polymerase chain reaction (PCR). (B) Effects of histone deacetylase 6 (HDAC6) siRNA on ER stress-related gene expressions in H226 cells. Endoplasmic reticulum (ER) stress loading after combined treatment with suberoylanilide hydroxamic acid (SAHA), bortezomib (BZ), and clarithromycin (CAM). (C) Cell growth inhibition of wild-type murine embryonic fibroblast (MEF) cell line and CHOP^−/−^ MEF cell line (16) after treatment with BZ in the presence/absence of SAHA (2.5 μM) and/or CAM (50 μg/ml) for 48 h. Viable cell numbers were assessed as described in Materials and methods. ^*^P<0.05 BZ/CAM/SAHA vs. BZ/SAHA and BZ/CAM.

Since aggresome is reported to form as an adaption process against overabundant intracellular unfolded or misfolded proteins, we hypothesized that HDAC6 inhibition with SAHA further enhances BZ/CAM-induced ER stress loading to the cells. As we expected, the expression levels of ER stress-related genes including *CHOP* were markedly pronounced in the presence of SAHA; the expression ratios of CHOP to untreated control cells increased to 5-fold by BZ/CAM treatment, and further increased to 25-fold by BZ/CAM/SAHA treatment in IM-9 cells (Fig. 7A). In H226 cells, similarly to SAHA, HDAC6 siRNA treatment enhanced BZ/CAM-induced CHOP expression compared with the cells treated with control siRNA (Fig. 7B). CHOP is an ER stress-related pro-apoptotic transcription factor which upregulates pro-apoptotic genes (e.g., *BIM*, *BAX*, *DR5*) and one of the critical molecules playing a role in the induction of apoptosis in response to ER stress (9,10). In addition, enhanced cytotoxicity was shown by a wild-type MEF cell line along with CHOP upregulation by the combination of the three reagents, whereas the CHOP^−/−^ MEF cell line derived from a *CHOP*-deficient mouse almost completely canceled this pronounced cytotoxicity (Fig. 7C). Therefore, the pronounced cytotoxicity and apoptosis in response to the SAHA/BZ/CAM combination treatment shown in Fig. 2A is strongly suggested to be due to the upregulation of CHOP in response to ER stress loading.

## Discussion

We showed that ER stress-mediated apoptosis in MM cells was potently enhanced by the simultaneous targeting of aggresome formation, proteasome, and the autophagy-lysosome system. Concomitant SAHA, BZ, and CAM treatment markedly enhanced CHOP induction via ER stress loading in MM cells (Fig. 7A), whereas a CHOP^−/−^ MEF cell line nearly completely cancelled the pronounced cytotoxicity in response to the SAHA/BZ/CAM combination treatment (Fig. 7C). These results strongly suggest that CHOP upregulation appears to be involved, at least in part, in the pronounced cytotoxicity shown in Fig. 2A. Our recent report also showed that combined treatment with SAHA/BZ/CAM exhibited potent cytotoxicity along with upregulation of CHOP in breast cancer cell lines (24). Since this enhanced cytotoxicity by ER stress loading is associated with inhibition of the BZ/CAM-induced aggresome formation (Figs. 4 and 5), induction of aggresome itself appears to function as cytoprotective against intracellular overloading of unfolded proteins as previously demonstrated in various neurodegenerative disorders (32,33).

In this study, we intentionally attempted to use the clinically approved drugs for blocking aggresome formation and the proteolytic pathways. Although exhibiting a potent inhibitory effect on HDAC6, SAHA (vorinostat) is a pan-HDAC inhibitor; therefore, the inhibitory effects on other HDACs cannot be completely excluded for the pronounced ER stress loading and apoptosis induction shown in Figs. 2A and 7A. However, tubacin, which is a specific inhibitor of HDAC6, also exhibited pronounced cytotoxicity (Fig. 3B), and knockdown of HDAC6 superimposed the effects of SAHA, such as suppression of vimentin-positive aggresome formation induced by BZ/CAM treatment (Fig. 4C and E), and further enhanced the ER stress-related gene upregulation along with the enhanced BZ/CAM treatment cytotoxicity (Figs. 3A and 7B). Therefore, HDAC6 appears to be a pivotal target for efficient ER stress loading under proteasome/autophagy inhibition. From the standpoint of ER stress loading, intracellular active protein synthesis appears to be theoretically an important factor for determining sensitivity. MM cell sensitivity to BZ/SAHA-induced cell death is regulated by Myc (34). Intracellular ER content, protein synthesis rates, percentage of aggresome-positive cells, and sensitivity to BZ/SAHA-induced cell death directly correlated with Myc expression. We previously reported that cycloheximide suppressed BZ/CAM-induced ER stress loading and cytotoxicity in MM cells (16). Thus, the potent effects shown in this study should depend in part on *de novo* protein synthesis. This may explain some differences among the MM cell lines, H226 cells, and MEF cell lines in terms of cytotoxicity and ER stress loading in response to these reagents shown in this study.

Along with vimentin-positive aggresome formation in the perinuclear region, Fig. 6 shows a number of autophagosomes and autolysosomes after BZ/CAM treatment. Electron microscopy demonstrated the clustering of autolysosomes in this area. These findings are in agreement with a recent study demonstrating that proteasome inhibition causes the formation of a zone around the centrosome where microtubular transport of lysosomes is suppressed, resulting in lysosome entrapment and accumulation (35). Interestingly, the authors of the previous study reported that the microtubule-dependent transport of other organelles, including autophagosomes, mitochondria and endosomes, is also blocked in this entrapment zone. Therefore, the perinuclear region stained with anti-vimentin Ab, where autolysosomes clustered along with the increased number of mitochondria, may in part overlap to ‘the entrapment zone’ (Figs. 4E and 6B) (35). It is also suggested that, upon proteasome inhibition, the targeting of aggregated proteins to aggresome is coordinated with lysosome positioning around the body to facilitate degradation via autophagy. However, our data showed that lysosomal clustering including autolysosome further became prominent by the combined treatment with CAM/BZ compared with that by the BZ treatment alone (Fig. 4F). Indeed, the addition of SAHA in the CAM/BZ culture medium suppressed lysosomal clustering in the perinuclear region; however, an increased number of cytosolic autolysosomes were still detectable (data not shown). This was possibly because of the suppression of lysosomal clearance upon autophagy inhibition by CAM. We and others have reported that macrolide antibiotics inhibit autophagy flux, although the precise molecular mechanism still remains to be clarified (16,17,36). The macrolide antibiotic BAF, which is a well-used autophagy inhibitor for *in vitro* experiments, was initially evaluated for its selective inhibition of a proton-pumping V-ATPase (37). BAF induces the disruption of vesicular proton gradients and raises the pH of acidic vesicles at nanomolar concentrations. This disruption of vesicular acidification in response to BAF appears to prevent autophagosome fusion with lysosomes, resulting in the inhibition of autophagy. It was also reported that treatment with AZM increased lysosomal pH in macrophages, which may lead to the inhibition of lysosomal hydrolases having an optimal low pH for their enzymatic activities (38). In this context, if CAM exerts the same effect on lysosomes, accumulation of autolysosomes by the suppression of lysosomal clearance itself appears to make ‘lysophagy’ apparent under proteasome inhibition. A similar phenomenon regarding the selective sequestration of damaged lysosome by autophagy was previously reported, in which under the condition of lysosomal damage induced by silica, monosodium urate, and a lysomotropic reagent, autophagy loss results in the *in vitro* inhibition of lysosomal biogenesis and the *in vivo* deterioration of acute kidney injury (39). Identification of the target molecules of macrolide antibiotics involved in autophagy flux inhibition, as well as the molecular mechanism of lysophagy including the recognition process of impaired lysosomes appears to be important issues that need to be clarified.

**Figure 8:**
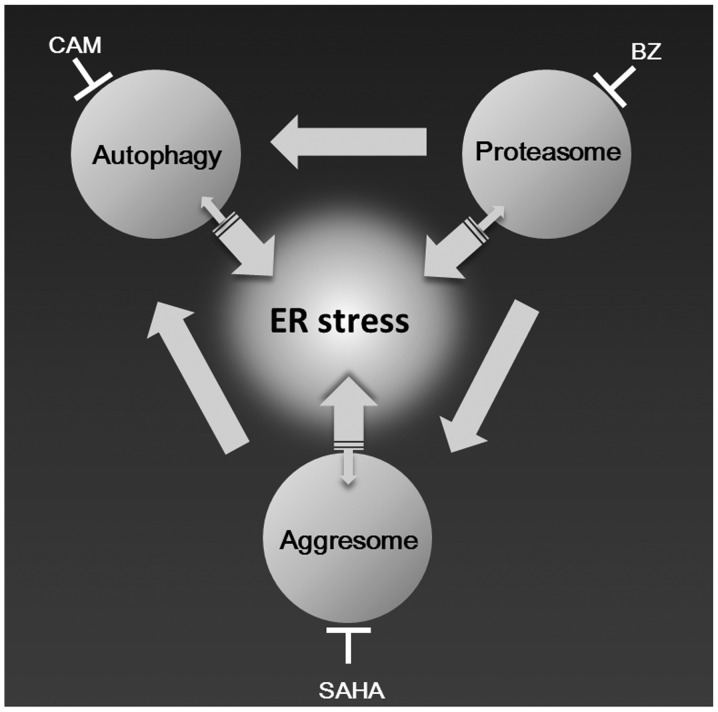
Proposed scheme for intracellular networks of unfolded protein processing and endoplasmic reticulum (ER) stress loading via systematic blockage of proteolytic pathways and aggresome formation.

Taking our data and accumulating lines of evidence together, we can draw an integrated network scheme among aggresome, proteasome, and autophagy as shown in Fig. 8 (9,10,13,14). Upon inhibition of proteasome and autophagy, overabundant ubiquitinated protein aggregates are transported to MTOC to form aggresomes. Under this condition, further inhibition of aggresome formation most efficiently induces ER stress loading in cells with a high protein synthesis rate such as MM cells. Thus, by combining clinically available drugs such as vorinostat, BZ, and CAM, this systematic strategy for blocking the processing of intracellular unfolded proteins appears to be applicable to refractory/relapsed MM patients.

## References

[1] Richardson PG, Barlogie B, Berenson J (2003). A phase 2 study of bortezomib in relapsed, refractory myeloma. N Engl J Med.

[2] San Miguel JF, Schlag R, Khuageva NK (2008). Bortezomib plus melphalan and prednisone for initial treatment of multiple myeloma. N Engl J Med.

[3] Richardson PG, Xie W, Jagannath S (2014). A phase 2 trial of lenalidomide, bortezomib, and dexamethasone in patients with relapsed and relapsed/refractory myeloma. Blood.

[4] Siegel DS, Martin T, Wang M (2012). A phase 2 study of single-agent carfilzomib (PX-171–003-A1) in patients with relapsed and refractory multiple myeloma. Blood.

[5] Dimopoulos M, Siegel DS, Lonial S (2013). Vorinostat or placebo in combination with bortezomib in patients with multiple myeloma (VANTAGE 088): a multicentre, randomised, double-blind study. Lancet Oncol.

[6] Orlowski RZ (2013). Novel agents for multiple myeloma to overcome resistance in phase III clinical trials. Semin Oncol.

[7] Martinon F (2012). Targeting endoplasmic reticulum signaling pathways in cancer. Acta Oncol.

[8] Hetz C, Chevet E, Harding HP (2013). Targeting the unfolded protein response in disease. Nat Rev Drug Discov.

[9] Tabas I, Ron D (2011). Integrating the mechanisms of apoptosis induced by endoplasmic reticulum stress. Nat Cell Biol.

[10] Verfaillie T, Salazar M, Velasco G, Agostinis P (2010). Linking ER stress to autophagy: potential implications for cancer therapy. Int J Cell Biol.

[11] Mizushima N, Levine B, Cuervo AM, Klionsky DJ (2008). Autophagy fights disease through cellular self-digestion. Nature.

[12] Mizushima N (2011). Autophagy in protein and organelle turnover. Cold Spring Harb Symp Quant Biol.

[13] Kirkin V, McEwan DG, Novak I, Dikic I (2009). A role for ubiquitin in selective autophagy. Mol Cell.

[14] Korolchuk VI, Menzies FM, Rubinsztein DC (2010). Mechanisms of cross-talk between the ubiquitin-proteasome and autophagy-lysosome systems. FEBS Lett.

[15] Kawaguchi T, Miyazawa K, Moriya S, Ohtomo T, Che XF, Naito M, Itoh M, Tomoda A (2011). Combined treatment with bortezomib plus bafilomycin A_1_ enhances the cytocidal effect and induces endoplasmic reticulum stress in U266 myeloma cells: Crosstalk among proteasome, autophagy-lysosome and ER stress. Int J Oncol.

[16] Moriya S, Che XF, Komatsu S, Abe A, Kawaguchi T, Gotoh A, Inazu M, Tomoda A, Miyazawa K (2013). Macrolide antibiotics block autophagy flux and sensitize to bortezomib via endoplasmic reticulum stress-mediated CHOP induction in myeloma cells. Int J Oncol.

[17] Komatsu S, Miyazawa K, Moriya S, Takase A, Naito M, Inazu M, Kohno N, Itoh M, Tomoda A (2012). Clarithromycin enhances bortezomib-induced cytotoxicity via endoplasmic reticulum stress-mediated CHOP (GADD153) induction and autophagy in breast cancer cells. Int J Oncol.

[18] Simms-Waldrip T, Rodriguez-Gonzalez A, Lin T, Ikeda AK, Fu C, Sakamoto KM (2008). The aggresome pathway as a target for therapy in hematologic malignancies. Mol Genet Metab.

[19] Kawaguchi Y, Kovacs JJ, McLaurin A, Vance JM, Ito A, Yao TP (2003). The deacetylase HDAC6 regulates aggresome formation and cell viability in response to misfolded protein stress. Cell.

[20] Ouyang H, Ali YO, Ravichandran M, Dong A, Qiu W, MacKenzie F, Dhe-Paganon S, Arrowsmith CH, Zhai RG (2012). Protein aggregates are recruited to aggresome by histone deacetylase 6 via unanchored ubiquitin C termini. J Biol Chem.

[21] Lee JY, Koga H, Kawaguchi Y (2010). HDAC6 controls autophagosome maturation essential for ubiquitin-selective quality-control autophagy. EMBO J.

[22] Fusco C, Micale L, Egorov M (2012). The E3-ubiquitin ligase TRIM50 interacts with HDAC6 and p62, and promotes the sequestration and clearance of ubiquitinated proteins into the aggresome. PLoS One.

[23] Yan J, Seibenhener ML, Calderilla-Barbosa L, Diaz-Meco MT, Moscat J, Jiang J, Wooten MW, Wooten MC (2013). SQSTM1/p62 interacts with HDAC6 and regulates deacetylase activity. PLoS One.

[24] Komatsu S, Moriya S, Che XF, Yokoyama T, Kohno N, Miyazawa K (2013). Combined treatment with SAHA, bortezomib, and clarithromycin for concomitant targeting of aggresome formation and intracellular proteolytic pathways enhances ER stress-mediated cell death in breast cancer cells. Biochem Biophys Res Commun.

[25] Olsen EA, Kim YH, Kuzel TM (2007). Phase IIb multicenter trial of vorinostat in patients with persistent, progressive, or treatment refractory cutaneous T-cell lymphoma. J Clin Oncol.

[26] Kavanaugh SM, White LA, Kolesar JM (2010). Vorinostat: a novel therapy for the treatment of cutaneous T-cell lymphoma. Am J Health Syst Pharm.

[27] Hubbert C, Guardiola A, Shao R, Kawaguchi Y, Ito A, Nixon A, Yoshida M, Wang XF, Yao TP (2002). HDAC6 is a microtubule-associated deacetylase. Nature.

[28] Haggarty SJ, Koeller KM, Wong JC, Grozinger CM, Schreiber SL (2003). Domain-selective small-molecule inhibitor of histone deacetylase 6 (HDAC6)-mediated tubulin deacetylation. Proc Natl Acad Sci USA.

[29] Garcia-Mata R, Bebök Z, Sorscher EJ, Sztul ES (1999). Characterization and dynamics of aggresome formation by a cytosolic GFP-chimera. J Cell Biol.

[30] Johnston JA, Ward CL, Kopito RR (1998). Aggresomes: a cellular response to misfolded proteins. J Cell Biol.

[31] Hung YH, Chen LM, Yang JY, Yang WY (2013). Spatiotemporally controlled induction of autophagy-mediated lysosome turnover. Nat Commun.

[32] Richter-Landsberg C, Leyk J (2013). Inclusion body formation, macroautophagy, and the role of HDAC6 in neurodegeneration. Acta Neuropathol.

[33] Hol EM, Fischer DF, Ovaa H, Scheper W (2006). Ubiquitin proteasome system as a pharmacological target in neurodegeneration. Expert Rev Neurother.

[34] Nawrocki ST, Carew JS, Maclean KH, Courage JF, Huang P, Houghton JA, Cleveland JL, Giles FJ, McConkey DJ (2008). Myc regulates aggresome formation, the induction of Noxa, and apoptosis in response to the combination of bortezomib and SAHA. Blood.

[35] Zaarur N, Meriin AB, Bejarano E, Xu X, Gabai VL, Cuervo AM, Sherman MY (2014). Proteasome failure promotes positioning of lysosomes around the aggresome via local block of microtubule-dependent transport. Mol Cell Biol.

[36] Nakamura M, Kikukawa Y, Takeya M, Mitsuya H, Hata H (2010). Clarithromycin attenuates autophagy in myeloma cells. Int J Oncol.

[37] Yamamoto A, Tagawa Y, Yoshimori T, Moriyama Y, Masaki R, Tashiro Y (1998). Bafilomycin A_1_ prevents maturation of autophagic vacuoles by inhibiting fusion between autophagosomes and lysosomes in rat hepatoma cell line, H-4-II-E cells. Cell Struct Funct.

[38] Renna M, Schaffner C, Brown K (2011). Azithromycin blocks autophagy and may predispose cystic fibrosis patients to mycobacterial infection. J Clin Invest.

[39] Maejima I, Takahashi A, Omori H (2013). Autophagy sequesters damaged lysosomes to control lysosomal biogenesis and kidney injury. EMBO J.

